# Irisin alleviates liver ischemia-reperfusion injury by inhibiting excessive mitochondrial fission, promoting mitochondrial biogenesis and decreasing oxidative stress

**DOI:** 10.1016/j.redox.2018.10.019

**Published:** 2018-10-24

**Authors:** Jianbin Bi, Jia Zhang, Yifan Ren, Zhaoqing Du, Qingshan Li, Yue Wang, Shasha Wei, Lifei Yang, Jingyao Zhang, Chang Liu, Yi Lv, Rongqian Wu

**Affiliations:** a National Local Joint Engineering Research Center for Precision Surgery & Regenerative Medicine, China; b Shaanxi Provincial Center for Regenerative Medicine and Surgical Engineering, China; c Institute of Advanced Surgical Technology and Engineering, China; d Department of Hepatobiliary Surgery, First Affiliated Hospital of Xi’an Jiaotong University, Xi’an, Shaanxi Province, China

**Keywords:** Hepatic I/R, Irisin, Mitochondrial homeostasis, Oxidative stress

## Abstract

Current management of liver ischemia-reperfusion (I/R) injury is mainly based on supportive care and no specific treatment is available. Irisin, a recently identified hormone, plays pivotal roles in energy expenditure and oxidative metabolism; however, it remains unknown whether irisin has any protective effects on hepatic I/R injury. In this study, we found that serum and liver irisin levels were markedly decreased at 24 h after hepatic I/R. Treatment with exogenous irisin improved liver function, reduced liver necrosis and cell apoptosis, and relieved inflammatory response after hepatic I/R. Meanwhile, exogenous irisin markedly inhibited mitochondrial fission related protein dynamin related protein 1 (drp-1) and fission 1 (Fis-1) expression in hepatic I/R. Additionally, treatment with exogenous irisin increased mitochondrial content and increased mitochondrial biogenesis related peroxisome proliferative activated receptor-γ (PPARγ) co-activator 1α (PGC-1α) and mitochondrial transcription factor (TFAM) expression. Furthermore, irisin decreased oxidative stress by upregulating uncoupling proteins (UCP) 2 expression in hepatic I/R. The results reveal that treatment with exogenous irisin alleviated hepatic I/R injury by restraining mitochondrial fission, promoting mitochondrial biogenesis and relieving oxidative stress. Irisin treatment appears to be a novel and promising therapeutic approach for hepatic I/R injury.

## Highlights

• Irisin protects hepatocytes against ischemia/reperfusion (I/R)-induced injury.
• Irisin inhibits excessive mitochondrial fission after hepatic I/R.
• Irisin promotes mitochondrial biogenesis after hepatic I/R.
• Irisin reduces oxidative stress after hepatic I/R.

## Graphical abstract

**Figure:**
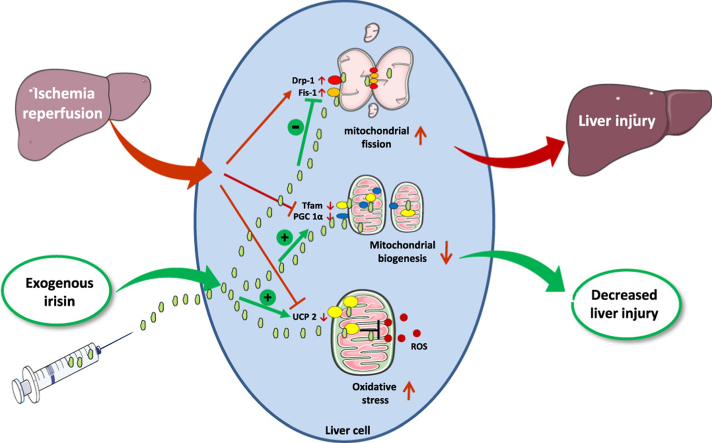

## 1 Introduction

Liver ischemia-reperfusion (I/R) injury is a major cause of hepatic dysfunction or failure following liver resection, liver transplantation, hemorrhagic shock, severe sepsis and so on [1]. Current management of hepatic I/R injury is mainly based on supportive care and no specific treatment is available.

Hypoxia and energy interruption under the ischemic condition lead to cell death. On the other hand, excessive reactive oxygen species (ROS), massive inflammatory mediators and mitochondrial dysfunction during the reperfusion trigger apoptosis and hepatic dysfunction [2], [3]. The balance between mitochondrial fission and fusion is essential for mitochondrial homeostasis [4]. In hepatic I/R, a decrease in the intracellular adenosine triphosphate (ATP) concentration increases mitochondrial fission by upregulating dynamin related protein 1 (drp-1) and fission 1 (Fis-1) expression to maintain mitochondria quantity [5]. However, excessive mitochondrial fission leads to mitochondrial fragmentation and activates the cell apoptosis pathway, which aggravates tissue injury [6]. In addition, mitochondrial biogenesis is severely decreased in hepatic I/R [7]. Peroxisome proliferative activated receptor-γ (PPARγ) co-activator 1α (PGC-1α), a mitochondrial biogenesis stimulator and a master regulator of ROS scavenging enzymes, is down-regulated in I/R [3], [8]. Decreased expression of uncoupling proteins (UCP) results in excessive ROS can also directly damage mitochondrial DNA (mtDNA), leading to mitochondrial dysfunction and subsequent depletion of ATP and cell death [9], [10].

Irisin, mainly secreted by the skeletal muscle and myocardium during exercise, is a secreted fragment of fibronectin type III domain containing 5 (FNDC5). It was initially discovered as a PGC1-α-dependent myokine and can convert the white adipose tissue to the brown adipose tissue [11]. Subsequent studies have shown that irisin could reduce oxidative/nitrative stress and protect endothelial cells in type 2 diabetes [12]. Additionally, irisin plays an important role in limb remote ischemic preconditioning (RIPC)-mediated lung protection via improvement of mitochondrial function [13]. However, the role of irisin in hepatic I/R injury remained unknown. We therefore hypothesized that irisin alleviates hepatic I/R injury by inhibiting excessive mitochondrial fission, promoting mitochondrial biogenesis and decreasing oxidative stress. The main purpose of the present study was to explore whether exogenous irisin could alleviate organ injury in a mouse model of hepatic I/R. Besides, the study also intended to clarify the effects of irisin on mitochondrial fission and fusion, mitochondrial content, mitochondrial biogenesis, ATP synthesis as well as oxidative stress in hepatic I/R.

## 2 Materials and methods

### 2.1 Experimental animals

Male wild-type C57BL/6J mice (6–8 weeks, 20–26 g) were purchased from the Experimental Animal Center of Xi’an Jiaotong University. The mice were housed in the Animal Feeding Center of Xi’an Jiaotong University Health Science Center (less than 5 mice per cage, 23 °C, 50% humidity, 12 h light/dark cycle). All animal experiments were performed in accordance with the guidelines of the China Council on Animal Care and Use and approved by the Institutional Animal Care and Use Committee of the Ethics Committee of Xi’an Jiaotong University Health Science Centeer, China (approval number: 2017-564).

### 2.2 Mouse model of hepatic I/R and experimental design

Partial (70%) liver warm I/R was performed as previous described [14]. Briefly, mice were anaesthetized with isoflurane. Partial (70%) liver arterial/portal venous blood was interrupted by an a traumatic clip cross the portal triad, above the right lateral lobe. After 60 min of ischemia, the clip was removed to allow reperfusion. Sham animals underwent the same surgical procedure without vascular occlusion. Mice were randomly allocated into the following groups (6 mice per group): 1) sham group, in which mice were given 0.5 ml saline after sham operation; 2) vehicle group, in which mice were intravenous administrated 0.5 ml saline at the beginning of reperfusion; and 3) irisin group, in which mice were intravenous administrated 250 μg/kg irisin (067-29A, Phoenix Pharmaceuticals, Inc. Burlingame, USA) in 0.5 ml saline at the beginning of reperfusion. At 24 h after reperfusion, mice were anaesthetized with isoflurane. Blood, liver, lung and kidney samples were collected.

### 2.3 Cell culture and hypoxia/reoxygenation (H/R)

HL-7702 cell line was purchased from the Procell Life Science&Technology Co.,Ltd. Cells were cultured in RPMI-1640 medium supplemented with 10% fetal bovine serum (FBS) and 100 units/ml penicillin/streptomycin mixture (Gibco, NY). Cell lines were incubated at 37 °C with 100% humidity in 5% CO_2_. In H/R model, HL-7702 cell line were cultured in RPMI-1640 medium (glucose/FBS free) and exposed to hypoxia condition (94% N_2_, 5% CO_2_, 1% O_2_) at 37 °C for 1 h. Then, the medium was changed to the normal and 10 ng/ml or 100 ng/ml irisin were treated for 2 h and 8 h.

### 2.4 Histological analysis

Liver and lung tissue samples were fixed in 4% paraformaldehyde for 48 h. Consecutive sections with 5 µm thickness were obtained from the paraffin. Hematoxylin and eosin (HE) staining of liver and lung tissues were performed. The histological changes were assessed in a blind manner by two researchers using a light microscope, and a representative field was chosen for application. Liver histological score was the sum of the individual score grades from 0, no; 1, mild; 2, moderate; and 3 severe for each of the following 6 items: cytoplasmic color fading, vacuolization, nuclear condensation, nuclear fragmentation, nuclear fading, and erythrocyte stasis, ranging from 0 to 18 [14]. Lung injury score was the sum of the individual score grades from 0, minimum; 1, mild; 2, moderate; 3, severe; and 4, maximum for each of the following 3 items: alveolar hemorrhage, infiltration or aggregation of inflammatory cells and thickness of the alveolar wall, ranging from 0 to 12 [15]. For transmission electron microscope, about 1 mm^3^ liver tissues were fixed in 2.5% glutaraldehyde and sample preparation was conducted as previous [16]. A representative field was chosen for application.

### 2.5 Enzyme-linked immunosorbent assays (ELISA)

The tumor necrosis factor a (TNF-α) ELISA kit (CSB-E04741m, Cusabio, Wuhan, China), cold-inducible RNA binding protein (CIRP) ELISA kit (CSB-EL005440MO, Cusabio, Wuhan, China) and irisin commercial ELISA kits (SEN576Mu, Cloud-Clone Corp USCN Life Science, Wuhan, China) were used for measuring the levels of serum TNF-α, CIRP and irisin according to the instructions of the manufacturer.

### 2.6 Measurement of liver function, lactate and ATP content

The serum alanine aminotransferase (ALT) assay Kit (C009-2, NanJing JianCheng Bioengineering Institute, Nanjing, China), aspartate aminotransferase (AST) assay Kit (C010-2, NanJing JianCheng Bioengineering Institute, Nanjing, China), lactic dehydrogenase (LDH) assay Kit (A020-2, NanJing JianCheng Bioengineering Institute, Nanjing, China) and lactate assay Kit (A019-2, NanJing JianCheng Bioengineering Institute, Nanjing, China) were used for measuring the levels of serum ALT, AST, LDH and lactate according to the instructions of the manufacturer. ATP Assay Kit was used for detecting ATP content (S0026, Beyotime Biotechnology, Shanghai, China).

### 2.7 Measurement of oxidative stress

Liver tissue homogenate was obtained and malonaldehyde (MDA) assay Kit (A003-1, NanJing JianCheng Bioengineering Institute, Nanjing, China), superoxide dismutase (SOD) assay Kit (A001–3, NanJing JianCheng Bioengineering Institute, Nanjing, China) and glutathione peroxidase activity (GSH-PX) assay Kit (A005, NanJing JianCheng Bioengineering Institute, Nanjing, China) were used for measuring the levels of liver MDA, SOD, GSH-PX according to the instructions of the manufacturer.

### 2.8 Apoptosis assay

Apoptotic cells were detected by Annexin V-FITC/PI Apoptosis Detection Kit (AD10, Dojindo laboratories, Shanghai, China) following the manufacturer's recommendations. The cells were analyzed with a flow cytometry (ACEA Biosciences, Inc.). The sum of early and late apoptotic cell percentage was defined as apoptotic cell percentage (n = 3 per group).

### 2.9 qPCR

The HL-7702 cell total RNA was isolated using TRIzol, and Drp-1, Fis-1 and PGC-1α mRNA expression were normalized to the β-actin mRNA. The primers were synthesized by Takara Biomedical Technology (Beijing) as follows: Homo sapiens Drp-1: Forward 5′- ACATCATCCAGCTGCCTCAAATC -3′, Reverse 5′- GTCTCCGGGTGACAATTCCAGTA -3′; Homo sapiens Fis-1: Forward 5′- GAACGAGCTGGTGTCTGTGGA-3′, Reverse 5′- AGCACGATGCCTTTACGGATG -3′; Homo sapiens PGC-1α：Forward 5′- AGCCAGCGTTCATGTTTGGTC -3′, Reverse 5′- TGCTAGCAAGTTTGCCTCATTCTC -3′; Homo sapiens β-actin: Forward 5′- TGGCACCCAGCACAATGAA -3′, Reverse 5′- CTAAGTCATAGTCCGCCTAGAAGCA-3′；mouse PGC-1α：Forward 5′- AAG ACG GAT TGC CCT CAT TT -3′, Reverse 5′- AGT GCT AAG ACC GCT GCA -3′; mouse β-actin: Forward 5′- GTG ACG TTG ACA TCC GTA AAG A -3′; Reverse, 5′- GTA ACA GTC CGC CTA GAA GCA C -3′). The relative levels were calculated using the Comparative-Ct Method (ΔΔCt method).

### 2.10 Analysis of mitochondrial DNA (mtDNA) content

mtDNA copy number was assessed by quantitative polymerase chain reaction (qPCR). The liver total DNA was isolated using DNeasy Blood and Tissue Kit (Qiagen, Valencia, CA) and mtDNA content was determined as mtDNA encoded NADH dehydrogenase-1 and normalized against the nuclear encoded POU class 5 homeobox 1 gene. The primers were synthesized by the Servicebio (Wuhan, China) as follows: NADH dehydrogenase-1: Forward 5′- GTGACGTTGACATCCGTAAAGA -3′, Reverse 5′- GTAACAGTCCGCCTAGAAGCAC -3′; POU class 5 homeobox 1: Forward 5′- AGAGTATGAGGCTACAGGGAC -3′, Reverse 5′- CAGAGCAGTGACGGGAACAGA -3′. The relative levels were calculated using the Comparative-Ct Method (ΔΔCt method).

### 2.11 Western blot analysis

The liver (ischemia part) protein was extracted and western blot was performed as previous described [17]. Proteins were separated by 7.5–15% sodium dodecyl sulphatepolyacrylamide gel electrophoresis (Bio-Rad, Hercules, CA). The PVDF membranes were blocked with 5% skim milk or BSA and incubated with primary antibodies, rabbit anti-irisin antibody (ab174833, Abcam, USA, 1:1000 dilution); rabbit anti-caspase-3 antibody (9662, Cell Signaling Technology, USA, 1:1000 dilution); rabbit anti-cleaved caspase-3 antibody (9661, Cell Signaling Technology, USA, 1:1000 dilution); rabbit anti-TFAM antibody (ab131607, Abcam, USA, 1:200 dilution); rabbit anti-Drp1 antibody (ab184247, Abcam, USA, 1:1000 dilution); rabbit anti-Fis1 antibody (GTX111010, GeneTex, USA, 1:250 dilution); rabbit anti-Mfn-2 antibody (ab124773, Abcam, USA, 1:5000 dilution); rabbit anti-UCP2 antibody (ab203244, Abcam, USA, 1:1000 dilution) and rabbit anti-β-actin antibody (4967, Cell Signaling Technology, USA, 1:1000 dilution). The membranes were incubated with secondary antibodies (HRP-conjugated Affinipure Goat Anti-Rabbit IgG(H+L), SA00001–2, Proteintech，USA, 1:2000 dilution) for 1 h at room temperature. The protein expression was detected using a chemiluminescence system (Bio-Rad, Hercules, CA). The protein quantification was performed using ImageJ2x software. The results are expressed as relative intensity of protein/β-actin.

### 2.12 Immunohistochemistry

Drp-1 and Fis-1 expression in the liver was detected by immunohistochemical staining. The detailed procedures were conducted as previously described [18]. The primary antibodies rabbit anti-Drp1 antibody (ab184247, Abcam, USA, 1:250 dilution); rabbit anti-Fis1 antibody (Santa Cruz Biotechnology, sc-376447, USA, 1:200 dilution) were incubated overnight at 4 °C, and a secondary antibody (SABC-HRP Kit with Anti-Rabbit IgG, P0615, Beyotime Biotechnology, Shanghai, China, 1:100 dilution) was incubated for 1 h at room temperature. The staining was assessed in a blind manner and a representative field was chosen for application. Immunohistochemical staining score was assessed by semi-quantitation grades from 0, negative; 1, mild; 2, moderate; and 3, severe, and extent of staining was graded based on the percentage of positive cells as follows: 0, negative; 1, 1–25%; 2, 26–50%; 3, 51–75%; and 4, 76–100%. The staining scores were the sum of the intensity and extent scores, ranging from 0 to 7.

### 2.13 Immunofluorescence staining

Immunofluorescence staining for MPO expression in the liver was performed as previous described [18] The primary antibody rabbit anti-MPO antibody (Santa Cruz Biotechnology, Inc., CA, diluted 1:200) were incubated overnight at 4°. Then the second antibody goat anti-rabbit antibody (Servicebio, China, diluted 1:300) were incubated 50 min at room temperature and counterstained with 4′-6-diamidino-2-phenylindole (DAPI). The sections were observed with a fluorescence microscope, and representative fields were chosen for application. The quantification of fluorescence intensity was performed using ImageJ2x software.

### 2.14 TUNEL, DHE and MitoTracker Red fluorescence staining

Briefly, cell death in paraformaldehyde fixed, paraffin embedded liver sections was detected by the transferase-mediated deoxyuridine triphosphate-biotin nick end labeling (TUNEL) (11684795910, Roche, Switzerland) according to the manufacturer's instructions. The sections were observed by a fluorescence microscope with excitation wavelength 480 nm and emission wavelength 530 nm, and representative fields were chosen for application. Dihydroethidium (DHE) dye (D7008, sigma-aldrich, USA) and MitoTracker Red CMXRos dye (M7512, Thermo Fisher Scientific, USA) was used for detection of mitochondria and ROS in live cells, respectively. For mitochondria detection, HL-7702 cells were incubated with MitoTracker Red CMXRos dye for 20 min at a concentration of 200 nM. The staining cells were observed by a confocal microscope with excitation wavelength 579 nm and emission wavelength 599 nm. For ROS detection, HL-7702 cells were incubated with DHE dye for 30 min at a concentration of 3 μM. The staining cells were observed by a confocal microscope (TCS SP8 STED 3×, Leica, Germany) with excitation wavelength 535 nm and emission wavelength 610 nm. The quantification of fluorescence intensity was performed using ImageJ2x software.

### 2.15 Statistical analysis

All the measurement data are expressed as the means ± standard error (SEM). The *t*-test or one way ANOVA was applied to analyze the differences between groups, using data statistics software SPSS 18.0. P < 0.05 represents a significant difference.

## 3 Results

### 3.1 Alterations in irisin levels after hepatic I/R

**Fig. 1:**
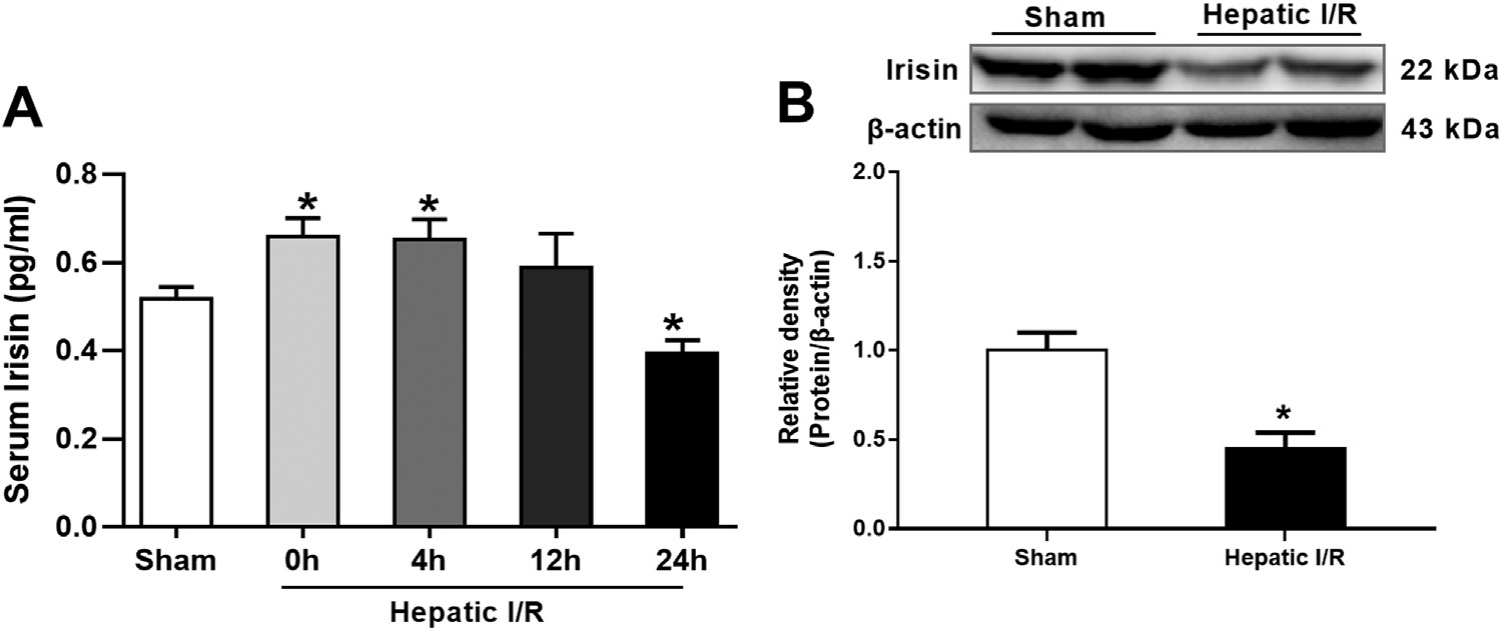
**Alterations in irisin levels after hepatic ischemia-reperfusion (I/R).** Partial (70%) liver arterial/portal venous blood was interrupted for 60 min. Blood samples were harvested at 0 h, 4 h, 12 h and 24 h after reperfusion. **A,** Serum irisin levels; **B**, Western blot analysis of liver irisin expressions. n = 6, mean ± SEM, *P < 0.05 versus sham group.

To investigate the changes of irisin secretion after hepatic I/R, irisin levels in the liver (ischemic part) and serum were measure in mice. As shown in Fig. 1A, serum levels of irisin increased significantly at the end of ischemia and 4 h after reperfusion. However, these increases faded at 12 h after reperfusion. At 24 h after reperfusion, serum levels of irisin were even significantly lower than those in sham operated animals (P < 0.05). Similarly, a markedly reduction in liver irisin levels was found at 24 h after hepatic I/R (Fig. 1B).

### 3.2 Treatment with exogenous irisin attenuates organ injury after hepatic I/R

**Fig. 2:**
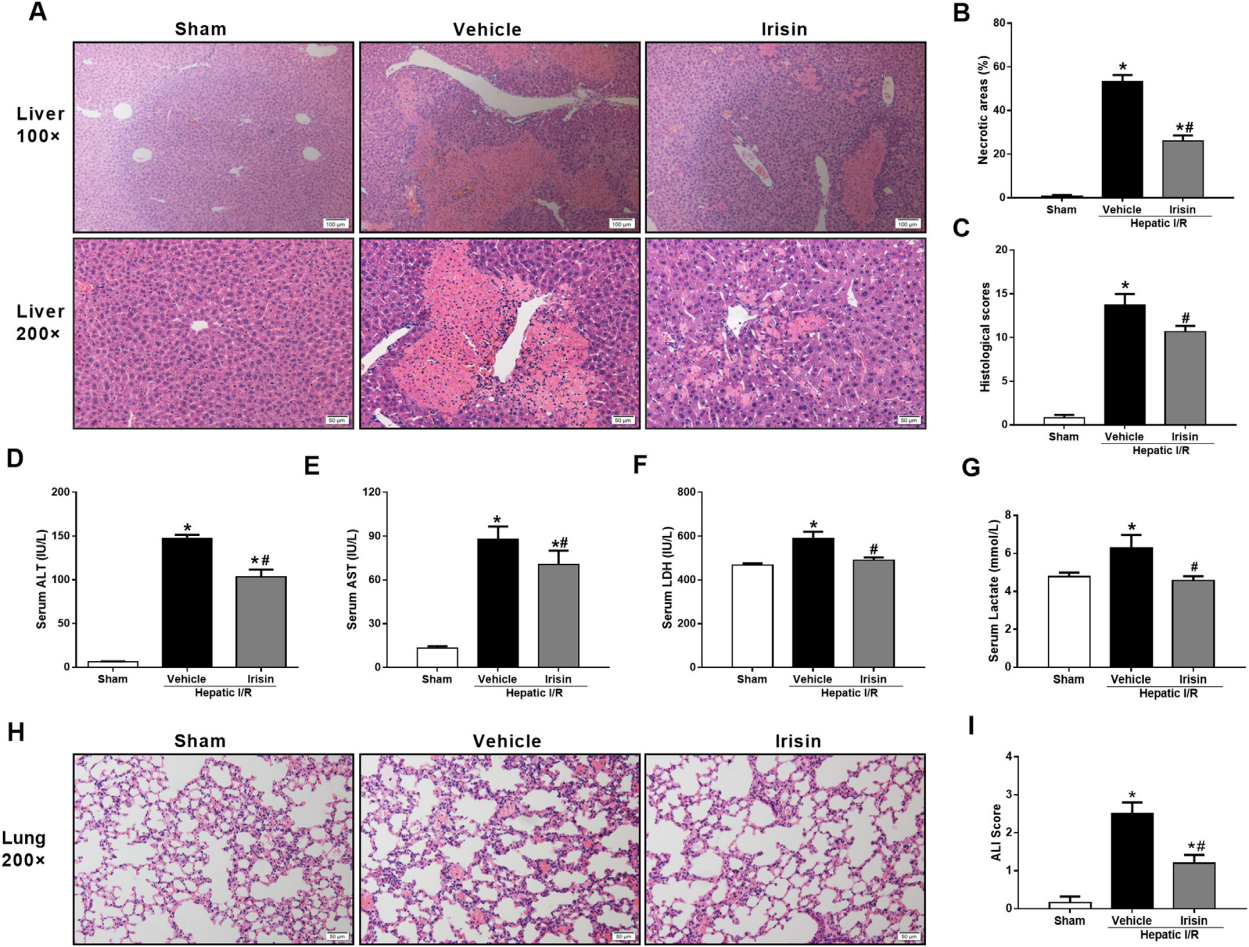
Treatment with exogenous irisin attenuates organ injury after hepatic ischemia-reperfusion (I/R). Irisin treatment in mice was conducted by intravenous administration (250 μg/kg, a single dose) at the beginning of reperfusion. The liver, lung and blood samples were harvested at 24 h after reperfusion. **A**, Hematoxylin and eosin staining (H&E) of representative liver sections (magnification 100× and 200×); **B,** Percentage of necrotic areas; **C,** Liver histological scores; **D-G**, The serum levels of alanine aminotransferase (ALT), aspartate aminotransferase (AST), lactic dehydrogenase (LDH) and lactate in each group, respectively；**H**, Hematoxylin and eosin staining (H&E) of representative lung sections (magnification 200×); **I,** Lung injury scores. n = 6, mean ± SEM, *P < 0.05 versus sham group, #P < 0.05 versus vehicle group.

To ascertain the effectiveness of exogenous irisin treatment on hepatic I/R, the liver injury was examined at 24 h after hepatic I/R. HE staining of the liver tissues revealed large areas of necrosis and inflammatory cell infiltration at 24 h after hepatic I/R in vehicle treated mice (Fig. 2A). Irisin treatment significantly alleviated the above changes. As indicated in Fig. 2B and C, both the size of necrosis areas and the hepatic injury score increased markedly at 24 h after hepatic I/R in vehicle-treated mice as compared with those in sham-operated mice. Administration of irisin significantly reduced the size of necrosis areas and the hepatic injury score at 24 h after hepatic I/R (p < 0.05). Consistent with the histologic data, serum levels of ALT, AST and LDH were significantly increased after hepatic I/R (Fig. 2D-F). Irisin treatment dramatically decreased the levels of these liver injury indicators at 24 h after hepatic I/R (P < 0.05). Besides, a decreased level of serum lactate was found in irisin-treated mice in contrast to vehicle-treated mice (Fig. 2G), indicating that irisin improves the metabolism condition after hepatic I/R. Hepatic I/R is often associated with lung injury [19], [20]. As shown in Fig. 2H and I, vehicle-treated mice had marked increase in lung injury scores at 24 h after hepatic I/R. Exogenous irisin treatment alleviated the hepatic I/R induced lung injury and the irisin group had significantly lower lung injury scores compared with the vehicle group at 24 h after hepatic I/R (p < 0.05).

### 3.3 Treatment with exogenous irisin decreases apoptosis after hepatic I/R

**Fig. 3:**
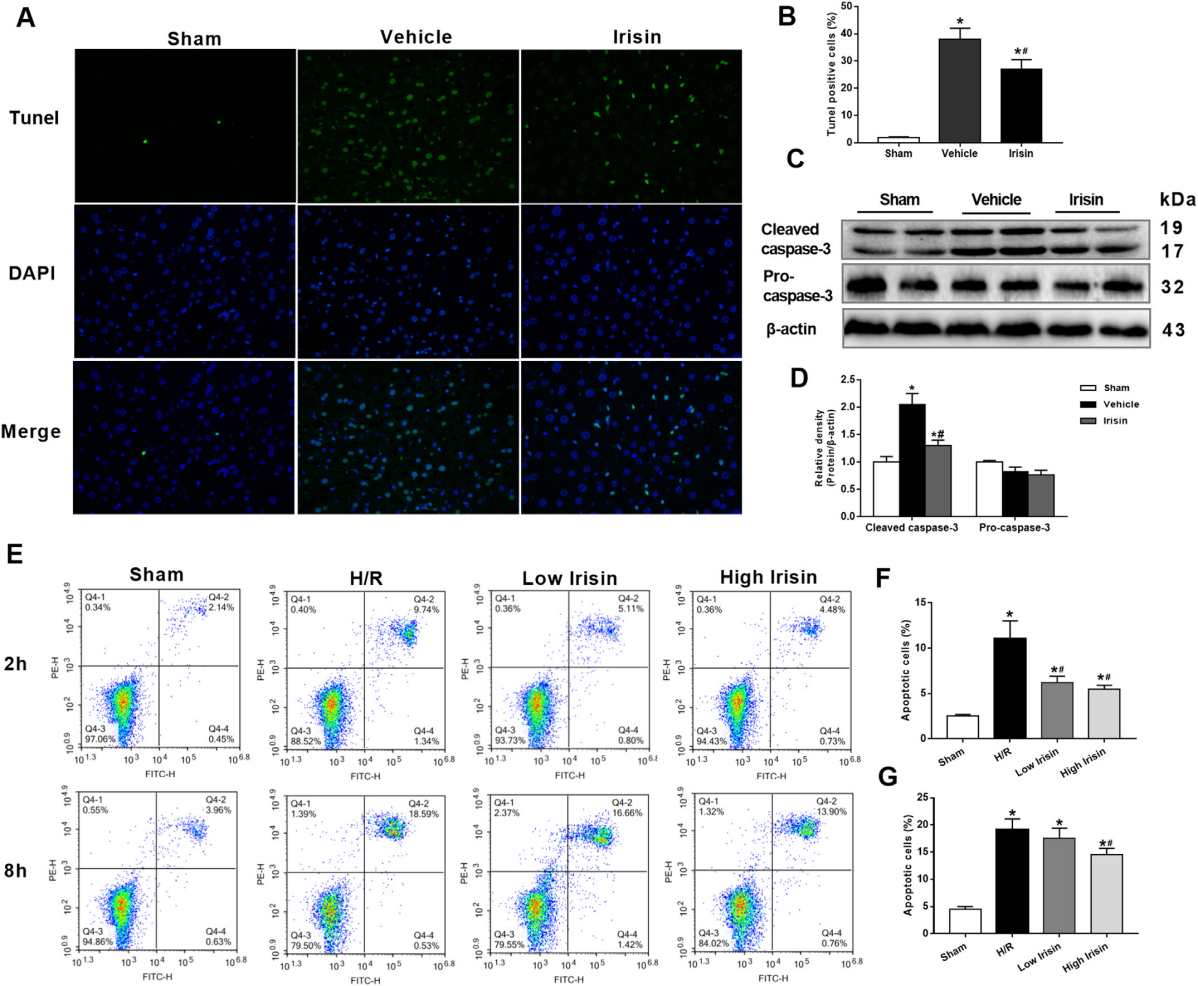
Treatment with exogenous irisin decreases apoptosis after hepatic ischemia-reperfusion (I/R). Irisin treatment in mice was conducted by intravenous administration (250 μg/kg, a single dose) at the beginning of reperfusion. The liver tissues were harvested at 24 h after reperfusion. **A**, transferase-mediated deoxyuridine triphosphate-biotin nick end labeling (TUNEL) fluorescence staining (green), the corresponding nuclear counterstaining (blue) and both channels merged of representative liver sections (magnification 400×); **B**, percentage of TUNEL positive cells; **C** and **D**, Western blot analysis of liver cleaved caspase 3 and pro-caspase 3 expression; n = 6, mean ± SEM, *P < 0.05 versus sham group, #P < 0.05 versus vehicle group. **E-G**, Flow cytometry analysis of HL-7702 cell apoptotic percentage at 2 h and 8 h after reoxygenation. n = 3, mean ± SEM, *P < 0.05 versus sham group, #P < 0.05 versus H/R group.

Compared with the sham operation group, livers from the vehicle-treated group exerted stronger positive TUNEL staining. Nevertheless, TUNEL positive cells were significantly reduced by treatment with exogenous irisin at the beginning of reperfusion (Fig. 3A and B). Meanwhile, western blot revealed that hepatic I/R markedly increased cleaved caspase-3 expression. However, a profound decrease was observed in the irisin group compared with the vehicle group (p < 0.05) (Fig. 3C and D), suggesting irisin plays an anti-apoptosis role in hepatic I/R injury in mice. The flow cytometry analysis indicated that hypoxia accelerated massive apoptosis of HL-7702 cells at 2 h and 8 h after reoxygenation. However, significant reductions of apoptotic cell percentage were observed in 10 ng/ml and 100 ng/ml irisin treatment at 2 h after reoxygenation. Meanwhile, at 8 h after reoxygenation, apoptotic cell percentages were markedly decreased at a concentration of 100 ng/ml irisin but not 10 ng/ml irisin (Fig. 3E-G).

### 3.4 Treatment with exogenous irisin downregulates inflammatory responses after hepatic I/R

**Fig. 4:**
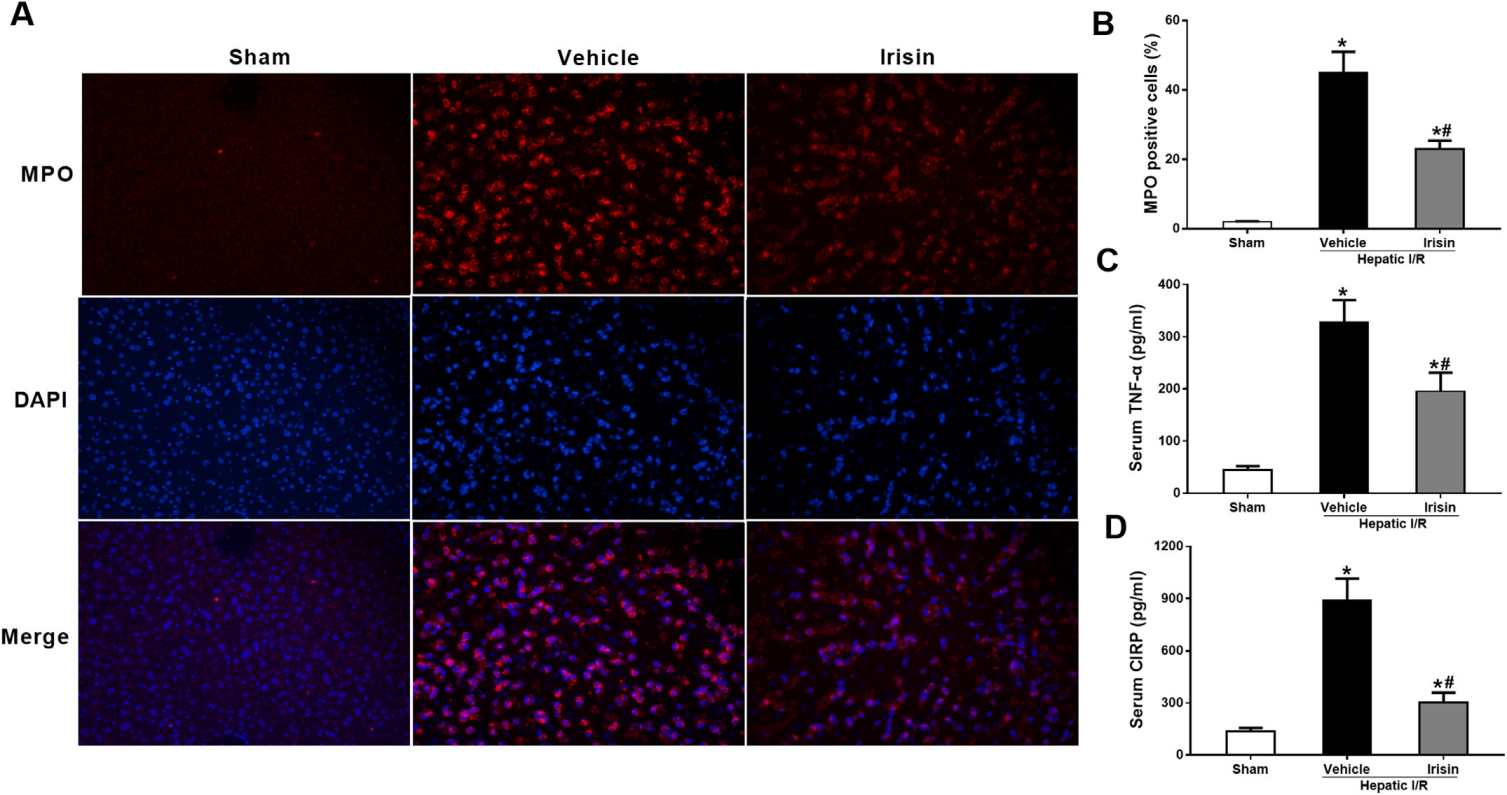
Treatment with exogenous irisin downregulates inflammatory responses after ischemia-reperfusion (I/R). Irisin treatment in mice was conducted by intravenous administration (250 μg/kg, a single dose) at the beginning of reperfusion. The liver tissues and blood samples were harvested at 24 h after reperfusion. **A**, Myeloperoxidase (MPO) immunofluorescence staining (green), the corresponding nuclear counterstaining (blue) and both channels merged of representative liver sections (magnification 400×); **B**, analysis of MPO fluorescence intensity; **C,** serum tumor necrosis factor α (TNF-α) levels; **D**, Serum cold-inducible RNA binding protein (CIRP) levels in each group. n = 6, mean ± SEM, *P < 0.05 versus sham group, #P < 0.05 versus vehicle group.

To determine the alterations in neutrophil infiltration, immunofluorescence staining of liver sections for MPO was performed. As shown in Fig. 4A and B, minimal immunostaining of MPO was detected in the liver of sham-operated animals. MPO immunostainings increased markedly at 24 h after reperfusion in vehicle-treated mice. Administration of irisin significantly reduced the MPO positive cells in the liver (P < 0.05). Proinflammatory cytokines TNF-α and CIRP play important roles in I/R injury [21], [22]. As shown in Fig. 4C and D, serum levels TNF-α and CIRP increased by 7.3 and 6.5 fold, respectively, at 24 h after reperfusion. Irisin treatment significantly decreased circulating levels of TNF-α and CIRP by 40.4% and 66.0%, respectively.

### 3.5 Treatment with exogenous irisin inhibits excessive mitochondrial fission after hepatic I/R

**Fig. 5:**
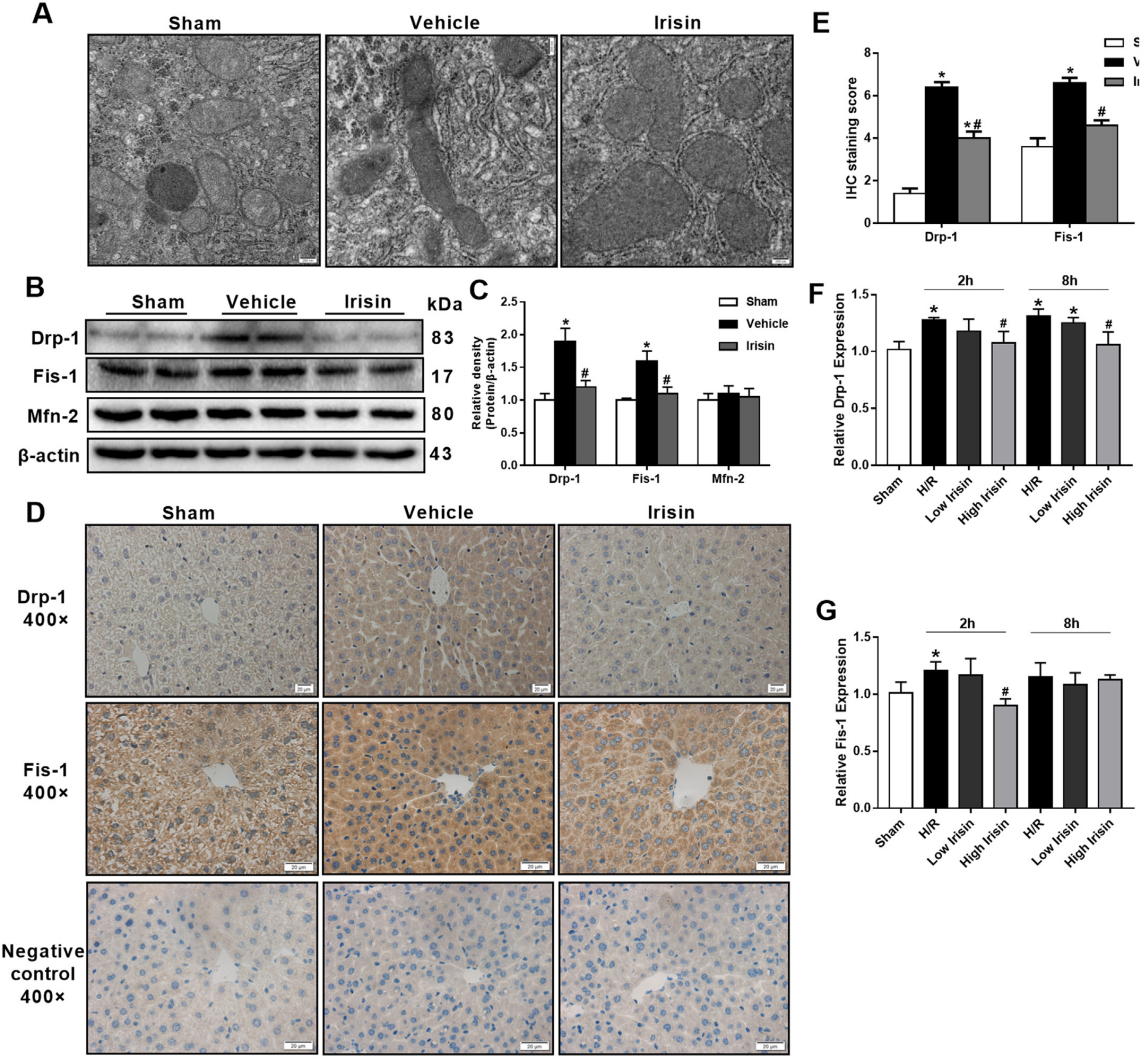
Treatment with exogenous irisin inhibits excessive mitochondrial fission after hepatic ischemia-reperfusion (I/R). Irisin treatment in mice was conducted by intravenous administration (250 μg/kg, a single dose) at the beginning of reperfusion The liver tissues were harvested at 24 h after reperfusion. **A**, Transmission electron microscope (TEM) of liver tissues (magnification 50,000×); **B** and **C,** Western blot analysis of liver dynamin related protein 1 (drp1), fission 1 (Fis-1) and mitofusin 2 (Mfn-2) expression. **D** and **E**，Immunohistochemistry and its semiquantitative analysis for evaluating the liver dynamin related protein 1 (drp1) and fission 1 (Fis-1) expression of representative liver sections (magnification 400×). n = 6, mean ± SEM, *P < 0.05 versus sham group, #P < 0.05 versus vehicle group. **F** and **G,** qPCR analysis of drp-1 and Fis-1 expression in HL-7702 cells at 2 h and 8 h after reoxygenation. n = 3, mean ± SEM, *P < 0.05 versus sham group, #P < 0.05 versus H/R group.

At 24 h after reperfusion, transmission electron microscope was conducted to observe liver mitochondrial changes in ischemic-reperfusion regions. We found that mitochondrial underwent extensive mitochondrial fission or fusion, mitochondria number was markedly decreased and mitochondria was swelling after hepatic I/R. Nevertheless, Irisin treatment group exhibited lower mitochondrial fission or fusion and more mitochondria though mitochondrial swelling was still existed (Fig. 5A). Imbalance of mitochondrial dynamics, including mitochondrial fission and fusion are initial process of cell apoptosis. Western blot indicated that Drp-1 and Fis-1, two mitochondrial fission related proteins, were significantly higher expressed in the vehicle group compared with those in the sham group (p < 0.05). However, Treatment with exogenous irisin markedly decreased the expression of Drp-1 and Fis-1 after hepatic I/R. The mitochondrial fusion related protein, Mfn-2, was not changed after hepatic I/R in both vehicle and irisin treated mice (Fig. 5B and C). Similarly, immunohistochemistry also indicated that treatment with exogenous irisin decreased Drp-1 and Fis-1 expression (Fig. 5D and E). Meanwhile, the qPCR results in vitro study showed that the expression of Drp-1 was reduced in 100 ng/ml irisin group in contrast to the H/R group in HL-7702 cells at 2 h and 8 h after reoxygenation. The Fis-1 expression was decreased in irisin group at 2 h but not 8 h after reoxygenation. Besides, 10 ng/ml irisin treatment showed no difference in Drp-1 and Fis-1 expression (Fig. 5F and G).

### 3.6 Treatment with exogenous irisin increases mitochondrial content by promoting mitochondrial biogenesis after hepatic I/R

**Fig. 6:**
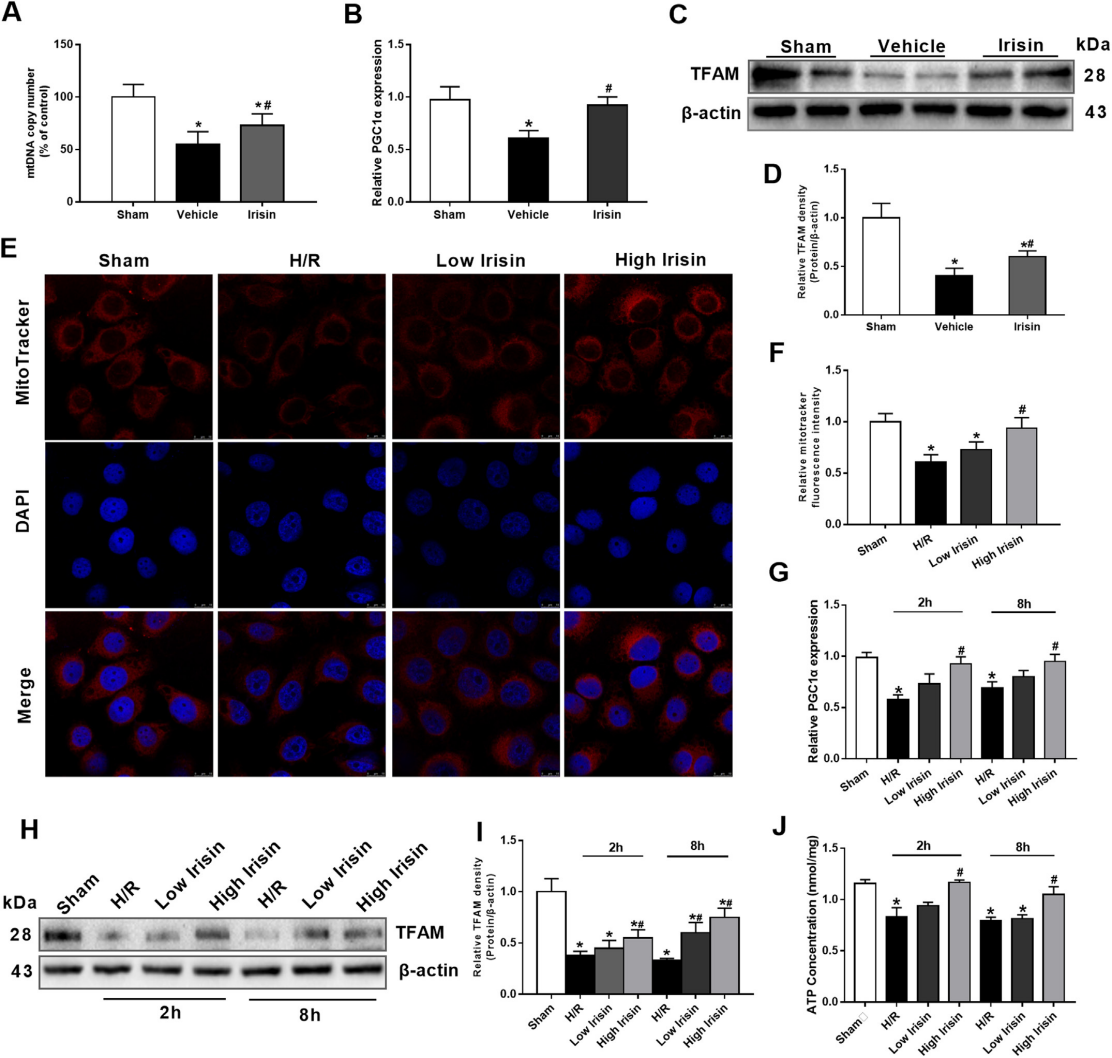
Treatment with exogenous irisin increases mitochondrial content by promoting mitochondrial biogenesis after hepatic ischemia-reperfusion (I/R). Irisin treatment in mice was conducted by intravenous administration (250 μg/kg, a single dose) at the beginning of reperfusion. The liver tissues were harvested at 24 h after reperfusion. **A,** Liver mtDNA copy numbers assessed by quantitative polymerase chain reaction (qPCR). **B**, qPCR analysis of liver peroxisome proliferative activated receptor-γ (PPARγ) co-activator 1α (PGC-1α) levels. **C** and **D,** Western blot analysis of liver transcription factor (TFAM) expression. n = 6, mea ± SEM, *P < 0.05 versus sham group, #P < 0.05 versus vehicle group. **E** and **F**, MitoTracker Red CMXRos fluorescence staining and its fluorescence intensity of HL-7702 cells at 2 h after reoxygenation; **G**, qPCR analysis of PGC-1α levels in HL-7702 cells at 2 h and 8 h after reoxygenation. **H** and **I,** Western blot analysis of TFAM expression in HL-7702 cells at 2 h and 8 h after reoxygenation; **J,** ATP content of HL-7702 cells at 2 h and 8 h after reoxygenation. n = 3, mean ± SEM, *P < 0.05 versus sham group, #P < 0.05 versus H/R group.

The decrease of mitochondrial content is a key factor in cell apoptosis after hepatic I/R. The mtDNA copy number, normalized against the nuclear DNA, was markedly reduced after hepatic I/R. However, treatment with exogenous irisin significantly improved the mtDNA copy number after hepatic I/R (Fig. 6A). PGC-1α and TFAM protein expressions are essential for mitochondrial biogenesis. As shown in the Fig. 6B, PGC-1α mRNA levels decreased markedly at 24 h after hepatic I/R. Treatment with irisin significantly upregulated PGC-1α gene expression in hepatic I/R mice. Although hepatic I/R decreased the TFAM protein expression in the liver, treatment with irisin exhibited significant increases in TFAM levels compared with those in vehicle-treated animals (Fig. 6C-D). In vitro study, mitoTracker fluorescence intensity was markedly increased in 100 ng/ml irisin group compared with those in the H/R group (Fig. 6E and F). Consistent with the results in vivo, exposure to hypoxia for 1 h reduced PGC-1α mRNA expression in HL-7702 cells by 42% and 31% at 2 h and 8 h after reoxygenation, respectively. At the concentration of 100 ng/ml, irisin significantly increased PGC-1α gene expression in hypoxia and reoxygenation treated HL-7702 cells (Fig. 6G). Similarly, the expression of TFAM was increased in 100 ng/ml irisin group in contrast to the H/R group at 2 h and 8 h after reoxygenation in HL-7702 cells (Fig. 6H and I). In addition, ATP content was measured and a significant increase of ATP content was found after 100 ng/ml irisin treatment at 2 h and 8 h after reoxygenation (Fig. 6J).

### 3.7 Treatment with exogenous irisin reduces oxidative stress after hepatic I/R

**Fig. 7:**
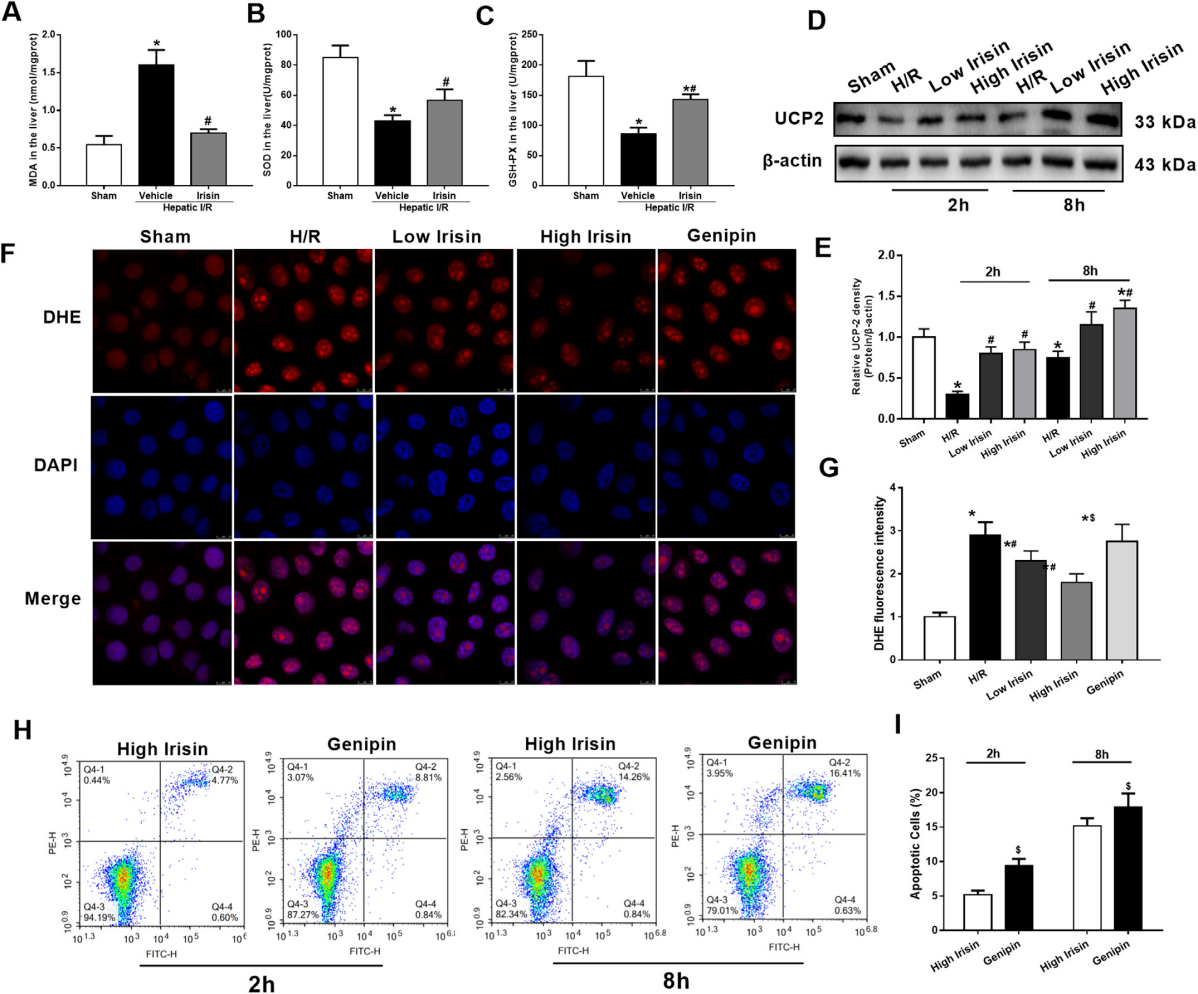
Treatment with exogenous irisin reduces oxidative stress after hepatic ischemia-reperfusion (I/R). Irisin treatment in mice was conducted by intravenous administration (250 μg/kg, a single dose) at the beginning of reperfusion. The liver tissues were harvested at 24 h after reperfusion. **A**-**C,** the malonaldehyde (MDA), superoxide dismutase (SOD) and glutathione (GSH) levels in the liver in each group, respectively. n = 6, mean ± SEM, *P < 0.05 versus sham group, #P < 0.05 versus vehicle group. **D** and **E,** Western blot analysis of UCP 2 expression in HL-7702 cells at 2 h and 8 h after reoxygenation; **F** and **G**, DHE fluorescence staining and its fluorescence intensity of HL-7702 cells at 2 h after reoxygenation; **H** and **I**, Flow cytometry analysis of HL-7702 cell apoptosis percentage at 2 h and 8 h after reoxygenation. n = 3, mean ± SEM, *P < 0.05 versus sham group, #P < 0.05 versus H/R group, $P < 0.05 versus high irisin group.

Oxidative stress is an initial and crucial factor in the process of hepatic I/R injury. Irisin treatment decreased the liver MDA level, while increased the levels of its reverse indicators, GSH-PX activity and SOD, compared with the vehicle-treated group (p < 0.05) (Fig. 7A-C). In HL-7702 cells, western blot showed a significant increase of UCP 2 expression after irisin treatment at 2 h and 8 h after reoxygenation (Fig. 7D and E). DHE fluorescence intensity was decreased after 100 ng/ml irisin administration. Genipin is a specific inhibitor of UCP 2. The results indicated that giving 100 ng/ml Genipin significantly reversed the therapeutic effects of irisin on decreasing ROS generation (Fig. 7F and G). In addition, Genipin treatment increased HL-7702 cell apoptosis after 100 ng/ml irisin treatment at 2 h and 8 h after reoxygenation (Fig. 7H and I).

## 4 Discussion

In the present study, we showed that treatment with exogenous irisin alleviated hepatic I/R injury in mice. The potential mechanisms might be related to irisin's role in inhibiting excessive mitochondrial fission, promoting mitochondrial biogenesis and decreasing oxidative stress. Irisin may meet the urgent medical need for preventing or minimizing hepatic I/R injury.

Since the discovery as a PGC1-α-dependent myokine in 2012, irisin has been given great expectations on the management of energy metabolism related diseases. Previous studies had proved that the levels of irisin expression in skeletal muscle upon exercise were decreased in obese rats compared with those in the healthy rats, and overexpression of irisin improved glucose/lipid metabolic metabolism in obesity [23], [24]. Additionally, a decreased level of irisin expression was discovered in patients with type 2 diabetes mellitus (T2DM), aging, renal diseases, and cardiovascular diseases [25], [26]. Circulating irisin levels were used as a marker for detecting early stage of cardiovascular and renal diseases [27], [28]. In the current study, we discovered that serum levels of irisin increased initially after hepatic I/R. However, at 24 h after reperfusion, both liver and serum levels of irisin were decreased. Given the fact that treatment with exogenous irisin ameliorated hepatic I/R injury, the early increase in irisin may be an endogenous protective mechanism for I/R injury, which exhausted at later stages. Interestingly, recent studies found that high irisin levels were positively related to insulin resistance, nonalcoholic fatty liver disease, subclinical atherosclerosis and associated with more severe NAFLD [29], [30]. However, increasing evidence has shown that irisin increases energy expenditure, improves insulin resistance, reduces hyperlipidemia and hyperglycemia, inhibits hepatic cholesterol synthesis, and ameliorates atherosclerosis [11], [23], [31], [32]. The increased release of irisin under such conditions could reflect a response to deterioration or a compensatory increase to overcome an underlying insulin resistance, nonalcoholic fatty liver disease or subclinical atherosclerosis [29].

Necrosis and apoptosis are two typical patterns of cell death as the consequence of hepatic I/R. Both necrosis and apoptosis can be caused by metabolic perturbation with ATP depletion [33]. In hepatic I/R, no-flow and low-flow hypoxia-induced mitochondria swollen releases cytochrome c, which facilitates caspase activation, leading to mitochondrial dysfunction and starts the cell apoptosis process [34]. Targeting the apoptosis process of cells like hepatocytes and stellate cells represents a therapeutic opportunity in hepatic I/R injury [35]. The previous studies proved irisin protects against endothelial injury and suppresses apoptosis in atherosclerosis [36]. Additionally, irisin protects human umbilical vein endothelial cell from high glucose-induced apoptosis [37]. In our study, treatment with exogenous irisin reduced the area of liver necrosis and decreased the apoptosis by inhibiting the activation of caspase-3, suggesting that irisin protects hepatocytes during I/R in mice.

Mitochondria constantly undergo fusion and fission to maintain mitochondrial homeostasis [38]. In hepatic I/R, the persistent metabolic stresses can lead to mitochondrial fission related protein Drp-1 and Fis-1 overexpression even in the absence of mitochondrial stress [5]. Excessive mitochondrial fission results in mitochondrial fragmentation and triggers cell apoptosis [39]. Our study showed that exogenous irisin treatment significantly decreased the expression of Drp-1 and Fis-1 after hepatic I/R. Irisin might inhibit apoptosis and increase mitochondrial content by decreasing excessive mitochondrial fission. In addition, mitochondrial fusion is a process of merging damaged mitochondria as a form of complementation, which is beneficial in maintaining functional mitochondria [36]. In hepatic I/R, autophagy is activated to remove damaged mitochondria and ATP synthesis is decreased due to the mitochondrial dysfunction [40]. In this study, no difference was found between the irisin and vehicle treated mice in mitochondrial fusion related Mfn-2. Thus, irisin is mainly related to mitochondrial fission not mitochondrial fusion after hepatic I/R.

One of the remarkable findings of the present study is that treatment with exogenous irisin increased mitochondrial content and promoting mitochondrial biogenesis after hepatic I/R in mice. PGC-1α is a major regulator in mitochondrial biogenesis and ROS metabolism. It is involved in the management of SOD-2 and GPx1 [8], [41]. Mitochondrial transcription factor A (TFAM), a downstream target of PGC-1α, regulates the replication and transcription of the mitochondrial genome [42]. In hypoxia conditions, mitochondrial biogenesis is rapidly increased to maintain mitochondrial content at the early stage to compensate the ATP insufficiency. However, the sustained hypoxia and ischemia results in reduction of PGC-1α and TFAM, leading the impaired mitochondrial biogenesis [43]. Increases in mitophagy and decreases in mitochondrial biogenesis contribute to a low level of mitochondrial content. Promoting mitochondrial biogenesis has been used as a strategy for alleviating hepatic I/R injury [44]. In our study, fluorescence intensity of MitoTracker Red CMXRos staining and the mtDNA copy number showed a serious decrease in mitochondrial content after hepatic I/R. However, exogenous irisin treatment significantly upregulated PGC-1α and TfFAM expression and increased mitochondrial content in the liver. Irisin might increase mitochondrial content in liver cells by up-regulating PGC-1α and TFAM expression.

Hepatic I/R leads to excessive ROS production [45]. ROS accumulation exceeds the endogenous anti-oxidant system and fuels oxidative stress. The ROS-dependent mitochondrial signaling pathway exerts crucial roles in the apoptotic or necrotic pathway of liver cells [46]. A lot of studies have confirmed that ROS scavenging is essential for relieving hepatic I/R injury [47], [48]. UCP 2 is activated by ROS to induce proton leak, which provides a negative feedback loop for mitochondrial ROS production [49]. A recent study demonstrated that irisin could reduce oxidative and nitrative stresses and protect endothelial cells in type 2 diabetes [12]. Our study also found that exogenous irisin treatment significantly decreased the levels of oxidation parameters such as ROS and MDA, while increased its reverse indicators, SOD and GSH after hepatic I/R. Furthermore, we found that irisin could markedly increase UCP 2 expression and inhibition of UCP 2 significantly reversed the protective effects of irisin on ROS generation and cell apoptosis. Thus, irisin might quench the oxidative stress by increase UCP 2 expression in hepatic I/R.

Some limitations need to be noted in the present study. Firstly, due to the low mortality in this model, the effect of irisin on hepatic I/R-related mortality was not observed in this study. Secondly, our present study mainly focused on the role of irisin in protecting mitochondrial homeostasis and quenching oxidative stress, other mechanisms of irisin in hepatic I/R injury need further exploration. Furthermore, despite exogenous irisin treatment achieving great therapeutic effects on hepatic I/R injury, our study was only based on basic experiments and prospective clinical studies are needed.

## 5 Conclusions

our studies demonstrate that exogenous irisin alleviates liver I/R injury by restraining mitochondrial fission, promoting mitochondrial biogenesis and relieving oxidative stress. Irisin treatment appears to be a novel and promising therapeutic approach for hepatic I/R injury.
